# Integrated multi-omics analysis reveals the molecular mechanism underlying poplar 107 rootstock–mediated regulation of *Populus tomentosa* scion growth

**DOI:** 10.1093/hr/uhag086

**Published:** 2026-03-11

**Authors:** Jianghao Wu, Yachao Ren, Chunyu Wang, Kaiyu Yang, Min Jiang, Jinmao Wang, Minsheng Yang

**Affiliations:** Institute of Forest Biotechnology, Forestry College, Agricultural University of Hebei, Baoding 071000, China; Hebei Key Laboratory for Tree Genetic Resources and Forest Protection, Baoding 071000, China; Institute of Forest Biotechnology, Forestry College, Agricultural University of Hebei, Baoding 071000, China; Hebei Key Laboratory for Tree Genetic Resources and Forest Protection, Baoding 071000, China; Institute of Forest Biotechnology, Forestry College, Agricultural University of Hebei, Baoding 071000, China; Hebei Key Laboratory for Tree Genetic Resources and Forest Protection, Baoding 071000, China; Institute of Forest Biotechnology, Forestry College, Agricultural University of Hebei, Baoding 071000, China; Institute of Forest Biotechnology, Forestry College, Agricultural University of Hebei, Baoding 071000, China; Hebei Key Laboratory for Tree Genetic Resources and Forest Protection, Baoding 071000, China; Institute of Forest Biotechnology, Forestry College, Agricultural University of Hebei, Baoding 071000, China; Hebei Key Laboratory for Tree Genetic Resources and Forest Protection, Baoding 071000, China; Institute of Forest Biotechnology, Forestry College, Agricultural University of Hebei, Baoding 071000, China; Hebei Key Laboratory for Tree Genetic Resources and Forest Protection, Baoding 071000, China

## Abstract

Grafting is widely used for asexual propagation and enhancing plant productivity; however, the molecular mechanisms underlying rootstock–scion interactions remain largely unclear. In this study, *Populus* *×* *euramericana* cv. ‘Neva’ (poplar 107) served as rootstock and *Populus tomentosa* ‘Yixian’ (*P. tomentosa*) as scion for grafting. By integrating physiological measurements, transcriptome sequencing [messenger RNA (mRNA) and long non-coding RNA (lncRNA)], widely targeted metabolomics, and mobile RNA identification revealed that heterografted scions had increased metabolites related to carbon fixation and metabolism, decreased metabolites associated with respiration and defense, and enhanced net photosynthetic rate, potentially promoting height growth; differential expression lncRNAs may represent an important molecular basis for accelerated scion growth; in scions, mobile mRNA-associated differential mRNAs primarily involved amino acid biosynthesis, while mobile lncRNA-associated ones focused on energy metabolism; no target mRNAs of mobile lncRNAs were found in the rootstock; both mRNAs and lncRNAs were transported in full-length and fragmented forms, and downward RNA transport showed a significant correlation with transcript abundance; in recipient tissues, mobile mRNAs were less abundant while mobile lncRNAs were more abundant than in donor tissues; the flavonoid biosynthesis pathway associated with mobile RNAs played an important role in rootstock-mediated scion growth, and kaempferol, as a key metabolite, reduced reactive oxygen species–induced damage, increased net photosynthetic rate, and promoted growth in *Nicotiana benthamiana*. This study provides an initial insight into the transport and regulatory patterns of mRNAs and lncRNAs in woody grafted plants, offering a theoretical basis for the rational application of grafting technology.

## Introduction

Grafting is an ancient breeding technique that joins two compatible plants through their vascular systems to transfer beneficial traits [1]. This technique is widely used in agricultural and forestry production, with notable examples including *Artemisia annua* rootstocks imparting superior ornamental traits to chrysanthemums [2], *Malus sieversii* rootstocks inducing early fruiting in apples [3], and Tifblue rootstocks enhancing fruit quality in blueberries [4]. These specific grafting combinations have stimulated deeper theoretical inquiry into the rootstock–scion interaction mechanism,aimed at the precise development of superior cultivars.

Rootstocks and scions can conduct substance exchange through the vascular system. The xylem is responsible for the transport of water and mineral salts, while the phloem can transport carbohydrates, amino acids, proteins, and hormones. This provides a prerequisite for rootstocks to confer desirable traits to scions. As demonstrated in the study by Liu *et al.* [5], the robust water absorption capacity of pumpkin rootstocks can enhance the transpiration rate of cucumber scions, thereby improving the cold resistance of cucumber plants. Additionally, Wang *et al.* [6] reported that the *Cyr1Ac* transgenic poplar Pb29 rootstocks are capable of transporting Cyr1Ac protein through the phloem, which in turn enhances the insect resistance of the scions. Rootstocks can also endow scions with desirable traits by modulating messenger RNA (mRNA) expression in scions. For example, grafting can alter the expression of mRNAs involved in hormone synthesis and signal transduction pathways in tomato scions, thereby enhancing their tolerance to salt stress [7]. In apple, rootstock-mediated grafting can regulate the expression of mRNAs associated with early flowering and hormone in scions, which in turn improves apple yield [8]. However, the mechanism underlying how rootstocks regulate mRNA expression changes in scions remains unclear. With the recognition that RNA is mobile and the subsequent discovery of a large number of RNAs (including mRNAs, sRNAs, and long non-coding RNAs **(**lncRNAs)] in the phloem, the role of mobile RNAs in signaling between rootstocks and scions has increasingly come into focus [9, 10]. Among these, mobile mRNAs have been the most extensively studied due to their role as the direct template for protein synthesis, which enables them to most likely synthesize proteins and exert functions in recipient tissues, along with their high sequence conservation and low sequencing cost. Currently, numerous studies have demonstrated that mobile mRNAs play crucial roles in regulating scion growth and development, as well as enhancing scion tolerance to abiotic stresses. For instance, the mobile *GAI* mRNA can regulate scion leaf size [11], while the mobile *CmoKARI1* mRNA modulates JA-Ile metabolism and improves scion cold tolerance [12].

In addition to mRNAs, plant genomes broadly transcribe numerous non-coding RNAs (ncRNAs), among which lncRNAs are generally defined as nucleotide sequences over 200 bp in length with no protein-coding capacity. Owing to their high proportion in ncRNAs and their ability to regulate gene expression through diverse and complex mechanisms, including but not limited to acting as signals, decoys, guides, or scaffolds in *cis* (at adjacent loci) or *trans* (at distal loci) to modulate chromatin states, transcriptional processes and post-transcriptional events [13], lncRNAs have attracted extensive research interest. However, the high species and tissue specificity of lncRNAs in terms of sequence conservation and expression patterns may be one of the reasons for the relative delay in their functional characterization [14]. With the in-depth study of ncRNAs, lncRNAs have been found to be mobile between rootstocks and scions. For example, in cucumber–watermelon graft combinations, 22 mobile lncRNAs were identified to translocate from rootstocks to scions, with their accumulation levels correlated with the degree of phosphorus stress [15]. Mobile lncRNAs can also move from rootstocks to scions and exert biological functions: in *Arabidopsis thaliana*, the lncRNA *ELENA1* translocates from rootstocks to scions under nitrogen deficiency, binds to MED19a, and competitively blocks the MED19a–ORE1 transcriptional complex, thereby delaying scion senescence [16]. These findings collectively indicate that mobile lncRNAs possess potential roles in signal transduction and functional regulation. To date, research on mobile lncRNAs has focused predominantly on herbaceous plants, with few studies conducted on woody species. Additionally, considering that even the mobility of homologous mRNAs differs between woody and herbaceous plants [17], and that mobile mRNAs also exert regulatory effects on gene expression in recipient tissues [5], it is highly necessary to simultaneously identify mobile mRNAs and lncRNAs in woody plants. This approach will not only advance the understanding of the transport and regulatory mechanisms of mobile RNAs in woody plants but also provide valuable genetic resources for genetic engineering-based breeding.

The superior traits conferred by rootstocks to scions rely not only on changes at the gene transcriptional level but also on alterations in metabolite profiles. For instance, variations in metabolites related to carbon–nitrogen metabolism, amino acid metabolism, and antioxidant metabolic pathways directly affect scion energy allocation and tissue development. Thus, metabolites serve as a crucial bridge connecting molecular signals and phenotypic traits. Moreover, relevant studies have demonstrated that rootstocks can reshape the metabolic network of scions to endow them with favorable traits: in grape graft combinations, rootstocks modify phenolic metabolites in the peel, thereby improving fruit quality [18]; in citrus graft combinations, rootstocks alter carbon metabolism, amino acid metabolism, and hormone levels in scions, enhancing their tolerance to abiotic stresses [19]. Therefore, metabolomic analysis, based on scion transcriptome sequencing and mobile RNA identification, will lay a solid foundation for in-depth exploration of the molecular mechanisms underlying the rootstock-mediated acquisition of superior traits in scions.

Poplar is an important timber and ecological tree species, from which a large number of clonal varieties have been selected, bred, and popularized. Grafting technology has become a crucial method for poplar asexual propagation—particularly the grafting propagation that combines difficult-to-root poplar varieties with easy-to-root varieties, which has been widely applied in production practice. *Populus × euramericana* cv. ‘Neva’ (poplar 107), a cultivar belonging to the section Aigeiros of the genus Populus, was introduced into China by the Chinese Academy of Forestry in 1984, it is characterized by rapid growth, strong adaptability, and high cutting survival rate. *Populus tomentosa* ‘Yixian’ (*P. tomentosa*) is an elite cultivar from the section Populus, featuring a straight trunk shape and excellent wood quality but low cutting survival rate. In production practice, using the easy-to-root poplar 107 as a rootstock enables the efficient propagation and popularization of *P. tomentosa*. However, the molecular mechanisms underlying how 107 rootstocks regulate the growth of *P. tomentosa* scions remain unclear. In this study, these two poplar varieties were used as materials for grafting experiments. By integrating physiological assays, transcriptomic analysis, metabolomic profiling, and identification techniques for mobile mRNAs and lncRNAs between rootstocks and scions, as well as functional verification of key metabolites, this study aims to preliminarily explore the molecular mechanisms underlying rootstock–scion interactions in woody plants and provide a scientific basis for the rational application of grafting technology in woody plant cultivation.

## Results

### The rootstock of 107 promotes the growth of mao scion

**Figure 1:**
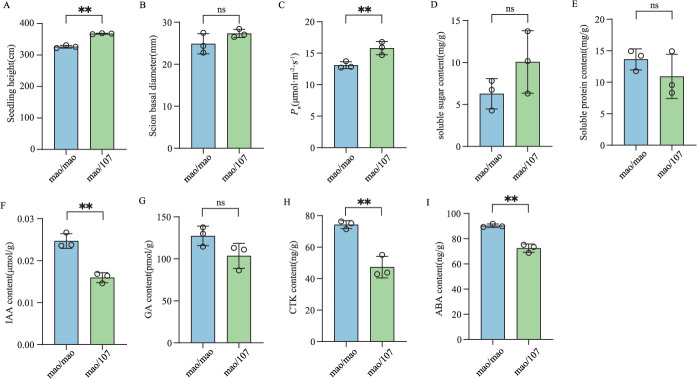
Determination of growth and physiological indices in mao scions. (A) Seedling height. (B) Scion basal diameter. (C) Net photosynthetic rate (*P_n_*). (D) Soluble sugar content. (E) Soluble protein content. (F) IAA content. (G) GA content. (H) CTK content. (I) ABA content. Bars represent the mean ± standard deviation (SD), and scatter points indicate individual raw data points. Differences between groups were analyzed using an independent samples *t*-test. ** denotes a significant difference (*P* < 0.05), while ‘ns’ indicates no significant difference.

To investigate the effect of the 107 rootstock on the growth of mao scions, growth-related traits (seedling height and basal diameter) and physiological indices (net photosynthetic rate, soluble sugar content, soluble protein content, and hormone contents) of mao scions were determined. Compared with self-grafted mao scions, heterografted mao scions showed significantly increased seedling height and net photosynthetic rate, along with significantly decreased contents of indole-3-acetic acid (IAA), cytokinins (CTKs), and abscisic acid (ABA). No significant differences were observed in basal diameter, soluble sugar content, soluble protein content, and GA content between the two graft combinations (Fig. 1).

### 107 Rootstock can significantly alter the metabolic profile of mao scions

**Figure 2:**
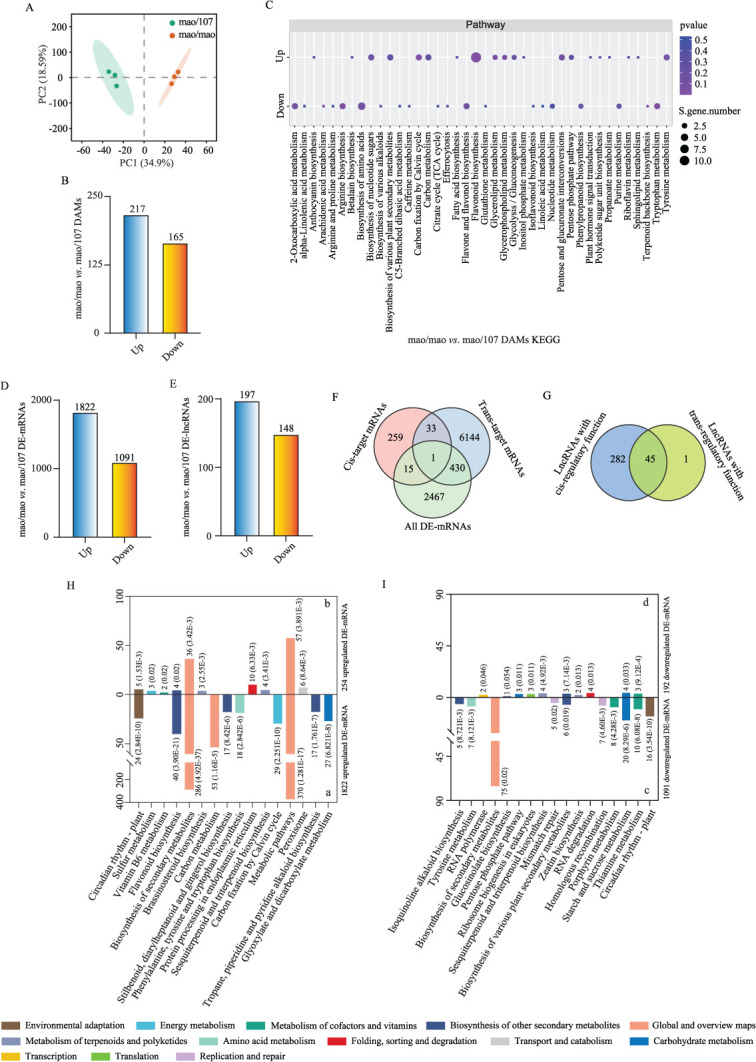
Heterografting induces DAMs and DE-RNAs in mao scions. (A) PCA result plot. (B) Plot of the number of upregulated and downregulated DAMs. (C) KEGG enrichment bubble plots for upregulated and downregulated DAMs; ‘Up’ and ‘Down’ represent upregulated and downregulated metabolites, respectively. The size and color of the dots indicate the number of metabolites and the enrichment level, respectively. (D) Plot of the number of upregulated and downregulated DE-mRNAs. (E) Plot of the number of upregulated and downregulated DE-lncRNAs. (F) Venn diagram of target mRNAs of DE-lncRNAs and DE-mRNAs. (G) Venn diagram of DE-lncRNAs with cis-regulatory roles and those with trans-regulatory roles. (H) KEGG enrichment bar plot of all upregulated mRNAs and all upregulated mRNAs associated with DE-lncRNAs. (I) KEGG enrichment bar plot of all downregulated mRNAs and all downregulated mRNAs associated with DE-lncRNAs. For the bar plots in H and I, the numbers above and below the bars represent the number of enriched mRNAs and the enrichment level, respectively.

A widely targeted metabolomics approach was used for the qualitative and quantitative analysis of metabolites in mao scions, aiming to investigate the effect of the 107 rootstock on mao scions at the metabolic level. In total, 3036 metabolites were identified from the mao scions. Principal component analysis (PCA) revealed that the three biological replicates within each group clustered closely together, indicating good sample reproducibility. PC1 explained 34.9% of the total variance and effectively discriminated between heterografted and self-grafted mao scions, confirming that the 107 rootstock indeed affected the metabolism of mao scions (Fig. 2A). Compared with self-grafted mao scions (mao/mao, control group), heterografted mao scions (mao/107, treatment group) exhibited 382 differential metabolites (DAMs), including 217 upregulated and 165 downregulated metabolites (Fig. 2B).

The upregulated and downregulated DAMs were subjected to Kyoto Encyclopedia of Genes and Genomes (KEGG) enrichment analysis separately. After sorting by enrichment degree (from high to low), the top 20 pathways for each group were selected for visualization. The results showed no overlap between the pathways of the two groups. For the upregulated DAMs, the enriched pathways related to energy fixation and metabolism included carbon fixation by Calvin cycle, carbon metabolism, glycolysis/gluconeogenesis, pentose phosphate pathway, and propanoate metabolism. Pathways associated with cell membrane and cell wall synthesis included glycerolipid metabolism, glycerophospholipid metabolism, sphingolipid metabolism, fatty acid biosynthesis, biosynthesis of nucleotide sugars, and pentose and glucuronate interconversions; pathways involved in signal transduction included plant hormone signal transduction and inositol phosphate metabolism; and pathways related to secondary metabolism included flavonoid biosynthesis, anthocyanin biosynthesis, betalain biosynthesis, biosynthesis of various plant secondary metabolites, and polyketide sugar unit biosynthesis. For the downregulated DAMs, the enriched pathways related to energy metabolism included citrate cycle (TCA cycle), 2-oxocarboxylic acid metabolism, and C5-branched dibasic acid metabolism; pathways associated with amino acid synthesis and metabolism included arginine biosynthesis, tryptophan metabolism, biosynthesis of amino acids, and arginine and proline metabolism; pathways involved in lipid defense signaling included arachidonic acid metabolism, alpha-linolenic acid metabolism, and linoleic acid metabolism; pathways related to secondary metabolism included terpenoid backbone biosynthesis, flavone and flavonol biosynthesis, caffeine metabolism, phenylpropanoid biosynthesis, biosynthesis of various alkaloids, and isoflavonoid biosynthesis; and the pathway associated with antioxidant stress was glutathione metabolism (Fig. 2C, Tables S1 and S2).

In summary, we hypothesize that 107 rootstocks may promote the growth of mao scions by enhancing their carbon fixation and carbon metabolism capacity, reducing defense and respiratory consumption, and providing more sufficient energy and structural substances for cell division and elongation.

### Differentially expressed lncRNAs and differentially expressed mRNAs and KEGG enrichment analysis in scions and rootstocks under heterografting

Transcriptome sequencing was performed on mao scions and 107 rootstocks from both self-grafted and heterografted combinations. For the six mao scion samples, the mapping rate of clean reads to the reference genome exceeded 91% in all cases, with the proportion of clean reads mapped to exons surpassing 77% and a Q30 value higher than 93%. For the 6107 rootstock samples, the mapping rate of clean reads to the reference genome was over 69.7%, the percentage of clean reads aligned to exons was more than 77.6%, and a Q30 value was above 93.5% (Table S3). These results indicate that the sequencing quality is high and suitable for subsequent downstream data analysis [20].

A total of 59 102 mRNAs were identified in the mao scions. Compared with self-grafted mao scions, heterografted mao scions had 2913 differentially expressed mRNAs (DE-mRNAs), including 1822 upregulated and 1091 downregulated ones (Fig. 2D). In parallel, 8061 lncRNAs were detected, among which 345 were DE-lncRNAs, with 197 upregulated and 148 downregulated ones (Fig. 2E). Of the identified DE-lncRNAs, 327 regulated 308 cis-target mRNAs, 16 of which were DE-mRNAs (Table S4). Additionally, 46 DE-lncRNAs regulated 6608 trans-target mRNAs, with 431 of these being DE-mRNAs (Table S5; Fig. 2F). Venn diagram analysis revealed that 45 DE-lncRNAs exerted both cis- and trans-regulatory effects on mRNAs (Fig. 2G). A total of 57 DE-lncRNAs were associated with the DE-mRNAs. Among these DE-lncRNAs, 38 were upregulated and 19 were downregulated, and the mRNAs they regulated included 254 upregulated and 192 downregulated ones.

To further clarify the functions of all DE-mRNAs and the DE-mRNAs regulated by DE-lncRNAs in heterografted mao scions, KEGG enrichment analysis was performed separately on all significantly upregulated and downregulated DE-mRNAs, and parallel analyses were conducted on the significantly upregulated and downregulated DE-mRNAs targeted by DE-lncRNAs (Tables S6–S9). The top 10 pathways for each group, ranked by enrichment degree from high to low, were subsequently visualized, and the results revealed that the enriched pathways of these two DE-mRNA sets were not completely identical (Fig. 2H and I). In energy metabolism-related pathways, the former (all DE-mRNAs) was predominantly specifically enriched in carbon metabolism, carbon fixation by Calvin cycle, and glyoxylate and dicarboxylate metabolism, while the latter (DE-mRNAs regulated by DE-lncRNAs) was mainly specifically enriched in sulfur metabolism and pentose phosphate pathway. In secondary metabolism-related pathways, the former was predominantly specifically enriched in stilbenoid, diarylheptanoid and gingerol biosynthesis, tropane, piperidine and pyridine alkaloid biosynthesis, and isoquinoline alkaloid biosynthesis, whereas the latter was specifically enriched in brassinosteroid biosynthesis, sesquiterpenoid and triterpenoid biosynthesis, glucosinolate biosynthesis, and zeatin biosynthesis. For pathways associated with metabolism of cofactors and vitamins, the former was specifically enriched in porphyrin metabolism, and the latter was mainly specifically enriched in vitamin B6 metabolism. Additionally, the former was uniquely enriched in amino acid metabolism-related pathways including phenylalanine, tyrosine and tryptophan biosynthesis, and tyrosine metabolism, as well as replication and repair-related pathways, such as mismatch repair and homologous recombination. In contrast, the latter was specifically enriched in pathways related to transcription, translation, folding, sorting and degradation, and transport and catabolism, including RNA polymerase, ribosome biogenesis in eukaryotes, RNA degradation, protein processing in endoplasmic reticulum, and peroxisome.

A total of 31 722 mRNAs were identified in the 107 rootstocks. Compared with self-grafted 107 rootstocks, heterografted 107 rootstocks had 389 DE-mRNAs, including 199 upregulated and 190 downregulated ones (Fig. S1A). In parallel, 5997 lncRNAs were detected, among which 37 were DE-lncRNAs, with 18 upregulated and 19 downregulated ones (Fig. S1B). Of the identified DE-lncRNAs, 34 regulated 32 cis-target mRNAs, with no differences in these cis-target mRNAs (Table S10); Additionally, five DE-lncRNAs regulated 2034 trans-target mRNAs, with 40 of these being DE-mRNAs (Table S11; Fig. S1C). Venn diagram analysis revealed that 5 DE-lncRNAs exerted both cis- and trans-regulatory effects on mRNAs (Fig. S1D). A total of five DE-lncRNAs were associated with the DE-mRNAs. Among these DE-lncRNAs, two were upregulated and three were downregulated, and the mRNAs they regulated included 25 upregulated and 15 downregulated ones.

To further clarify the functions of all DE-mRNAs and the DE-mRNAs regulated by DE-lncRNAs in heterografted 107 rootstocks, KEGG enrichment analysis was performed separately on all significantly upregulated and downregulated DE-mRNAs, and parallel analyses were conducted on the significantly upregulated and downregulated DE-mRNAs targeted by DE-lncRNAs (Tables S12–S15). The top 10 pathways for each group, ranked by enrichment degree from high to low, were subsequently visualized, and the results revealed that the enriched pathways of these two DE-mRNA sets were not completely identical (Fig. S1E and F). In energy metabolism-related pathways, the former (all DE-mRNAs) was predominantly specifically enriched in carbon metabolism, carbon fixation by Calvin cycle, photosynthesis, and starch and sucrose metabolism, while the latter (DE-mRNAs regulated by DE-lncRNAs) was mainly specifically enriched in glyoxylate and dicarboxylate metabolism and sulfur metabolism. In secondary metabolism-related pathways, the former was primarily specifically enriched in terpenoid backbone biosynthesis, whereas the latter was specifically enriched in phenylpropanoid biosynthesis. In amino acid metabolism-related pathways, the former was mainly specifically enriched in cyanoamino acid metabolism, and the latter was predominantly specifically enriched in glutathione metabolism and tryptophan metabolism. For pathways associated with metabolism of cofactors and vitamins, the former was primarily specifically enriched in porphyrin metabolism, while the latter was mainly specifically enriched in ubiquinone and other terpenoid–quinone biosynthesis. Additionally, the latter was uniquely enriched in motor proteins related to cell motility, peroxisome associated with transport and catabolism, purine metabolism linked to nucleotide metabolism, and linoleic acid metabolism related to lipid metabolism.

In conclusion, the DE-lncRNAs in mao scions and 107 rootstocks under heterografting may represent one of the crucial factors regulating the DE-mRNAs. In heterografted mao scions, the DE-mRNAs involved in energy metabolism, hormone synthesis, as well as transcription and translation pathways associated with DE-lncRNAs may provide an important molecular basis for the rapid growth of scions.

### Mobile mRNAs and lncRNAs exert distinct regulatory effects on scions

To further investigate the regulatory mechanisms underlying DE-mRNAs in the mao scions and 107 rootstocks under heterografting, rootstock–scion mobile mRNAs and lncRNAs were identified. If mobile mRNAs exhibit differential expression in the donor tissues, such differential expression is considered to be associated with their mobility. Because orthologous genes are defined as genes derived from the same ancestral gene in different species and generally retain identical or similar functions [21], the differential expression of the best reciprocal orthologous mRNAs in recipient tissues compared with their corresponding mobile mRNAs in the donor tissue suggests that such differential expression is associated with mRNA translocation [5]. In contrast, lncRNAs exhibit relatively low sequence conservation; therefore, if the target mRNAs of differentially expressed mobile lncRNAs in the donor tissues, as well as the target mRNAs corresponding to all donor-derived mobile lncRNAs in the recipient tissues, show differential expression, mobile lncRNAs are considered to be associated with the differential expression of rootstock–scion mRNAs.

**Figure 3:**
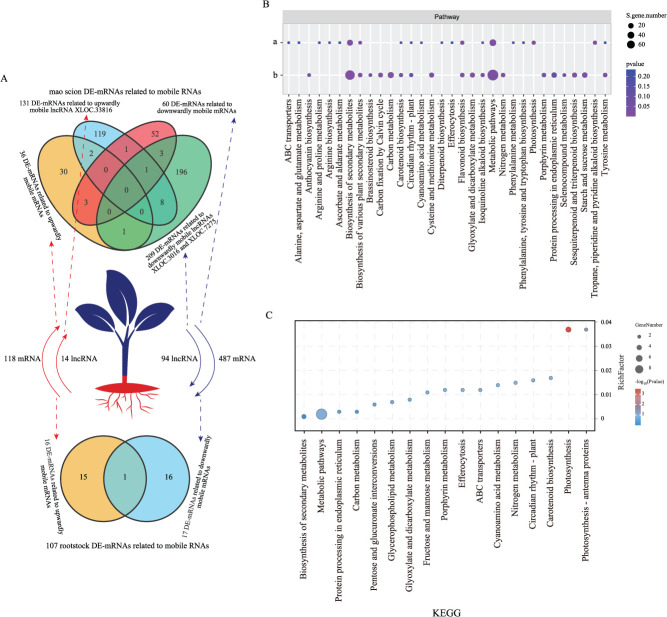
Number and KEGG enrichment of DE-mRNAs associated with rootstock–scion mobile RNAs. (A) Number of mobile mRNAs and mobile lncRNAs between rootstocks and scions, as well as the numbers of DE-mRNAs associated with them; Red and blue dashed lines indicate DE-mRNAs associated with upward- and downward-mobile mRNAs or lncRNAs, respectively. (B) KEGG enrichment bubble plot of DE-mRNAs in the mao scions associated with mobile mRNAs and mobile lncRNAs, respectively; a represents the enriched pathways of DE-mRNAs associated with mobile mRNAs, and b represents those associated with mobile lncRNAs. (C) KEGG enrichment bubble plot of DE-mRNAs in 107 rootstock associated with mRNA mobility.

A total of 118 mRNAs were identified as mobilized upward from the 107 rootstocks in heterografts. Of these, 16 mRNAs were significantly upregulated compared with those in the 107 rootstocks of self-grafts (Table S16). In the mao scions, 114 best reciprocal orthologous mRNAs corresponding to these mobilized mRNAs from the 107 rootstocks were identified, among which 36 exhibited significant upregulation (Table S17). In addition, 14 lncRNAs were identified as mobilized upward from the 107 rootstocks to scions in heterografts; however, none of these lncRNAs showed differential expression compared with those in the 107 rootstocks of self-grafts (Table S18). Among them, only XLOC.33816 was predicted to regulate 2609 trans-target mRNAs in the mao scions. Of these target mRNAs, 131 were associated with the DE-mRNAs in the heterografted mao scions, including 77 significantly upregulated and 54 significantly downregulated (Table S19; Fig. 3A).

A total of 487 mRNAs were identified as mobilized downward from the mao scion in heterografts; Of these, 60 mRNAs exhibited significant differences compared with those in the mao scions of self-grafts, including 49 significantly upregulated and 11 significantly downregulated (Table S20). In the 107 rootstocks, 427 best reciprocal orthologous mRNAs corresponding to these mobilized mRNAs from the mao scions were identified, of which 17 showed significant differential expression in the heterografted 107 rootstocks, including 16 significantly upregulated and 1 significantly downregulated (Table S21). In addition, 94 lncRNAs were identified as mobilized downward from the mao scion in heterografts. Among these, 13 lncRNAs were significantly differentially expressed compared with those in the mao scions of self-grafts, including 10 significantly upregulated and 3 significantly downregulated (Table S22). Among these, XLOC.3016 and XLOC.7275 were predicted to regulate 3583 trans-target mRNAs in the mao scions. Of these target mRNAs, 209 showed significant differential expression, including 150 significantly upregulated and 59 significantly downregulated (Table S23). Notably, no trans-target mRNAs were predicted for the downwardly mobilized lncRNAs in the 107 rootstocks (Fig. 3A).

The top 20 KEGG pathways enriched for DE-mRNAs associated with mRNA mobility and those associated with lncRNA mobility in the heterografted mao scions were distinct. DE-mRNAs associated with mobile mRNAs were primarily enriched in amino acid metabolism, such as alanine, aspartate and glutamate metabolism, arginine and proline metabolism-related pathways, such as alanine, aspartate, and glutamate metabolism; arginine and proline metabolism; cyanoamino acid metabolism, and phenylalanine, tyrosine, and tryptophan biosynthesis. In contrast, DE-mRNAs associated with mobile lncRNAs were primarily enriched in energy metabolism-related pathways, such as carbon fixation by Calvin cycle, carbon metabolism, nitrogen metabolism, and starch and sucrose metabolism. Additionally, DE-mRNAs associated with both mobile mRNAs and mobile lncRNAs were co-enriched in secondary metabolism–related pathways (including carotenoid biosynthesis, flavonoid biosynthesis, and isoquinoline alkaloid biosynthesis), as well as in the environmental adaptation–related pathway circadian rhythm–plant (Fig. 3B). Notably, several KEGG pathways enriched for DE-mRNAs in 107 rootstocks associated with mobile mRNAs were consistent with those identified in scions, including carotenoid biosynthesis, circadian rhythm–plant, nitrogen metabolism, and carbon metabolism. These findings suggest that mobile RNA–mediated DE-mRNAs enriched in shared pathways between rootstocks and scions may play a crucial role in coordinated scion–rootstock growth and in the process by which rootstocks confer superior agronomic traits to scions (Fig. 3C).

### Upward- and downward-mobile RNAs exhibited distinct correlations with transcript abundance

**Figure 4:**
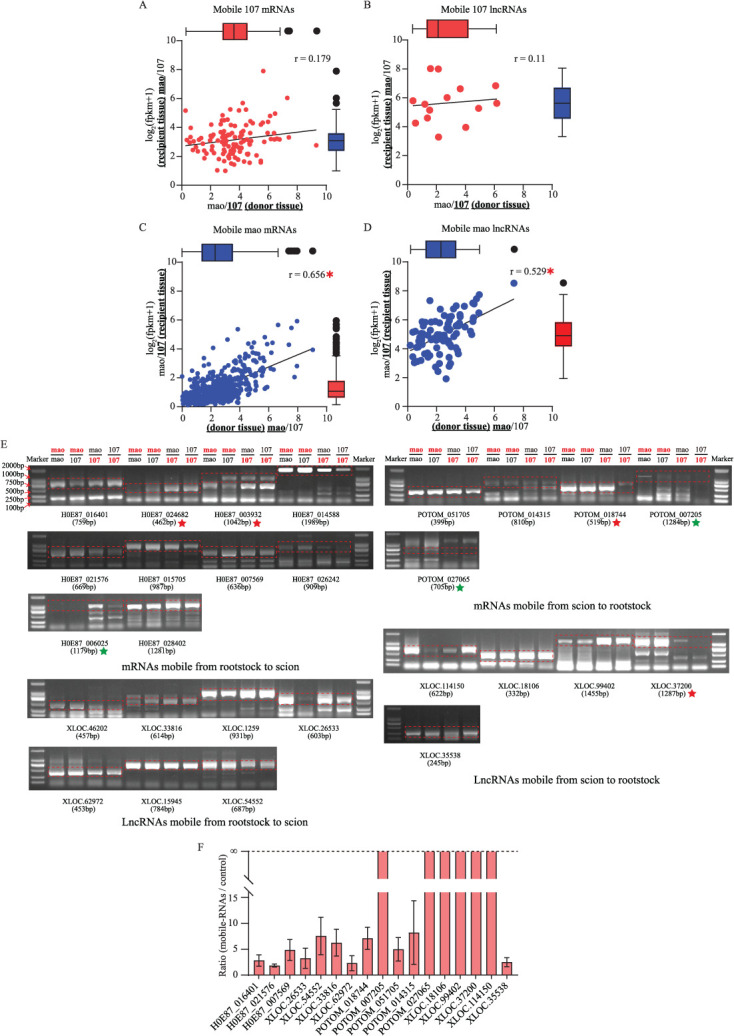
Expression abundance, correlation analysis, and validation of rootstock–scion mobile RNAs. (A) Expression abundances and correlation of upwardly mobilized mRNAs in the 107 rootstocks and the mao scions. (B) Expression abundances and correlation of upwardly mobilized lncRNAs in the 107 rootstocks and the mao scions. (C) Expression abundances and correlation of downwardly mobilized mRNAs in the 107 rootstocks and the mao scions. (D) Expression abundances and correlation of downwardly mobilized lncRNAs in the 107 rootstocks and the mao scions. In panels A–D, underscores on the *x*-axis indicate donor tissues of mobile RNAs, while underscores on the *y*-axis indicate recipient tissues; Correlations between variables were analyzed using Pearson's correlation coefficient, and * indicates a significant correlation; Boxplots were drawn using Tukey's method. (E) Validation of the accuracy of scion-rootstock mobile RNAs by RT-PCR. A 2000-bp DNA marker was used in all experiments; ☆ indicates cases where a band was present in the recipient tissue but absent in the control, while ☆ indicates cases where a band was absent in both the recipient tissue and the control. The red text denotes the tissue where the target band was detected; the scion is shown above the horizontal line, and the rootstock is shown below it. (F) RT-qPCR results, the *y*-axis represents the ratio of the relative expression level of the RNA in the recipient tissue to that in the control.

We employed scatter plots, box plots, and correlation analysis to investigate the relationship between the expression abundance of mobile RNAs in donor and recipient tissues. In terms of abundance, the overall expression abundance of mobile mRNAs in donor tissues was slightly higher than that in recipient tissues, whereas the overall expression abundance of mobile lncRNAs in donor tissues was lower than that in recipient tissues. Correlation coefficients showed that the abundances of downward-mobile mRNAs and downward-mobile lncRNAs in the mao scions were significantly correlated with those in the 107 rootstocks. In contrast, the abundances of upward-mobile mRNAs and upward-mobile lncRNAs in the 107 rootstocks showed no significant correlation with those in the mao scions (Fig. 4A–D). These results indicate that the mechanisms underlying upward and downward RNA mobility may be distinct.

### RT-PCR and RT-qPCR validation for the accuracy of rootstock–scion mobile RNAs

Twenty-seven mobile RNAs were randomly selected for RT-PCR identification. Among them, clear bands of only four RNAs were detected at the target positions in recipient tissues, with no bands observed in the controls. For 20 RNAs, bands were present in all tissues, making it impossible to distinguish between homologous sequences. Additionally, three RNAs showed bands in donor tissues but no bands in recipient tissues and controls (Fig. 4E). To further validate the accuracy of the above RNA (mRNAs and lncRNAs) movement results, first, Sanger sequencing was performed on the four mobile RNAs that showed bands in the recipient tissue but no bands in the control. The results indicated that all 4 RNAs were transported in full length (Fig. S2). Subsequently, 20 RNAs were randomly selected from the 27 mobile RNAs for RT-qPCR validation. It was found that the ratio of the relative expression level in the recipient tissue to that in the control exceeded 2-fold for 17 of these RNAs; among them, 6 RNAs had an infinite ratio, resulting in a validation accuracy of 85% (Fig. 4F). Among these six mobile RNAs, *POTOM_007205* and *POTOM_027065* showed no bands in the recipient tissue and the control; *XLOC.18106*, *XLOC.99402*, and *XLOC.114150* exhibited bands in both the recipient tissue and the control, while *XLOC.37200* displayed a band in the recipient tissue but no band in the control. These results demonstrate that full-length and fragmented mRNAs and lncRNAs undergo movement between mao scions and 107 rootstocks.

The above three full-length mobile mRNAs included two mRNAs from the 107 rootstocks (*H0E87_024682* and *H0E87_003932*) and 1 mRNA from the mao scion (*POTOM_018744*). Among them, the best homologous mRNA of *H0E87_024682* in *A. thaliana* was *AT1G56300*, annotated as DJC53, which is involved in the regulation of plant circadian rhythm [22, 23]. The best homologous mRNA of *H0E87_003932* in *A. thaliana* was *AT5G42800*, annotated as dihydroflavonol 4-reductase, which promotes anthocyanin biosynthesis [24]. For *POTOM_018744*, its best homologous mRNA in *A. thaliana* was *AT5G64040*, annotated as PSAN. This mRNA is involved in the formation of the photosystem I-N subunit, which can improve plant photosynthetic efficiency and is of great importance for promoting plant growth [25]. The above RNAs with an infinite ratio included two mRNAs and three lncRNAs (undetermined mobile sequences) from mao scions, plus one lncRNA (determined mobile sequence) from the same source. Among these RNAs, the best homologous mRNA of *POTOM_007205* in *A. thaliana* was *AT1G60420*, annotated as NRX1, which is associated with enhancing plant reactive oxygen species (ROS) resistance [26]. For *POTOM_027065*, its best homologous mRNA in *A. thaliana* was *AT4G37560*, annotated as IAMH2, which can catalyze the conversion of indole-3-acetamide (IAM) to IAA [27]. Among the pathways significantly enriched by all target mRNAs of the four lncRNAs, the pathway with the largest number of enriched mRNAs was carbon metabolism (Table S24). These results further indicate that the mobile RNAs between scion and rootstock may play an important regulatory role in the growth of grafted plants.

### The flavonoid biosynthesis pathway was significantly affected in the heterografted mao scions

**Figure 5:**
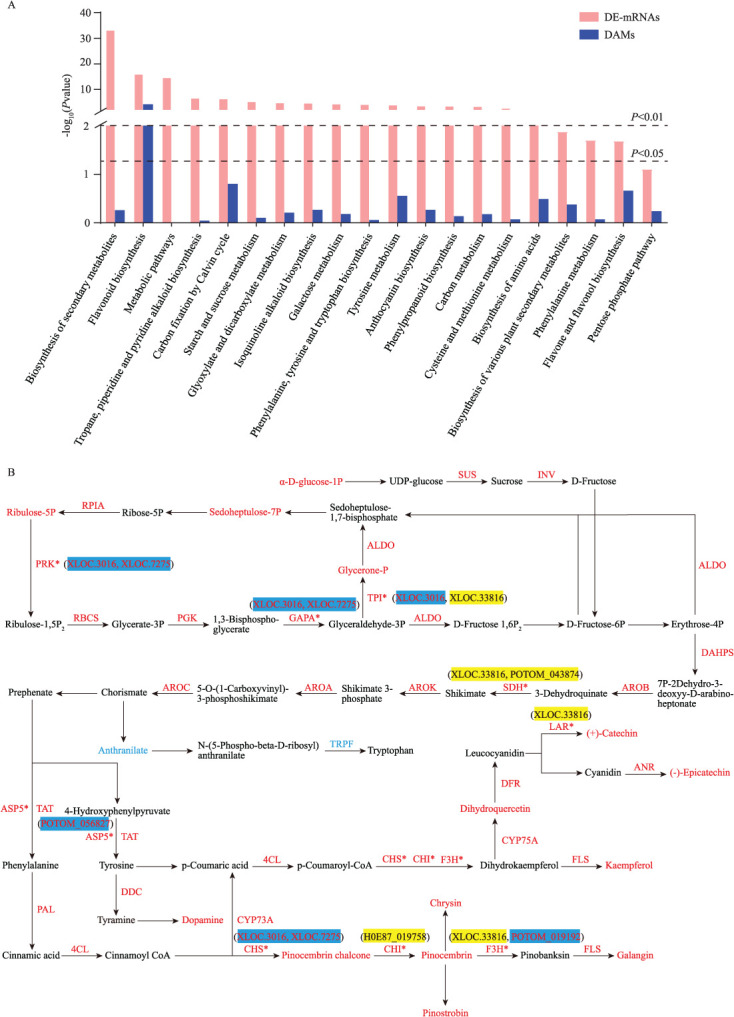
Integrated transcriptomic and metabolomic analysis. (A) KEGG pathways co-enriched by differential mRNAs and metabolites in the scions of heterografted mao. (B) Mechanism diagram of pathways related to energy metabolism, amino acid metabolism, and flavonoid biosynthesis. Red text outside parentheses indicates significantly upregulated differential mRNAs or metabolites, and cyan text indicates significantly downregulated ones; * denotes differential mRNAs related to mobile RNAs (*P* < 0.05). Blue-background red text within parentheses represents downwardly mobile mRNAs and lncRNAs associated with differential mRNAs in the mao scions, while yellow-background black text represents upwardly mobile mRNAs and lncRNAs associated with differential mRNAs in themao scions. INV, beta-fructofuranosidase; RPIA, ribose 5-phosphate isomerase A; ALDO, fructose-bisphosphate aldolase; RBCS, ribulose-bisphosphate carboxylase small chain; PGK, phosphoglycerate kinase; DAHPS, 3-deoxy-7-phosphoheptulonate synthase; AROC, chorismate synthase; AROA, 3-phosphoshikimate 1-carboxyvinyltransferase; AROK, shikimate kinase; SDH, shikimate dehydrogenase; AROB, 3-dehydroquinate synthase; 4CL, 4-coumarate—CoA ligase; FLS, flavonol synthase; CYP75A, flavonoid 3′,5′-hydroxylase; DFR, bifunctional dihydroflavonol 4-reductase/flavanone 4-reductase; ANR, anthocyanidin reductase; PAL, phenylalanine ammonia-lyase; DDC, aromatic-L-amino-acid decarboxylase; CYP73A, trans-cinnamate 4-monooxygenase.

To comprehensively understand the molecular mechanism by which the 107 rootstocks promotes the growth of the mao scions, an integrated transcriptomic and metabolomic analysis was performed (Table S25). The results showed that among the top 20 pathways co-enriched by DE-mRNAs and DAMs, only the flavonoid biosynthesis pathway was the only one with significant enrichment of both (Fig. 5A). The transition from primary metabolism to secondary metabolism is energy-driven. Given that the 107 rootstocks significantly influenced the expression of mRNAs in pathways related to carbon fixation and carbon metabolism in the mao scions, and these pathways were also enriched with DAMs, erythrose-4-phosphate is considered a key intermediate linking the carbon fixation in the Calvin cycle pathway to the biosynthesis of aromatic amino acids via D-Fructose 6P. Furthermore, phenylalanine and tyrosine serve as important precursors for flavonoid biosynthesis. Accordingly, we integrated selected branches from the following six pathways—carbon fixation by the Calvin cycle, starch and sucrose metabolism, phenylalanine, tyrosine and tryptophan biosynthesis, phenylpropanoid biosynthesis, flavonoid biosynthesis, and isoquinoline alkaloid biosynthesis—all of which ranked among the top 20 enriched pathways, to construct a mechanistic diagram. In these pathways, all DE-mRNAs and DAMs were significantly upregulated except those related to tryptophan biosynthesis, which were significantly downregulated. Notably, several key changes were identified as critical factors driving the alterations in metabolites accumulation and mRNA expression within the flavonoid biosynthesis pathway: in the Calvin cycle, ribulose-5-phosphate (Ru5P)—a molecule involved in CO₂ acceptor regeneration—and the expression levels of mRNAs associated with the phosphoribulokinase (PRK) were significantly upregulated; in the starch and sucrose metabolism pathway, the substrate α-d-glucose-1P (associated with UDP-glucose synthesis) and mRNAs encoding enzymes, such as sucrose synthase (SUS) and invertase (INV), responsible for sucrose synthesis and hydrolysis, were significantly upregulated; similarly, in the phenylalanine, tyrosine, and tryptophan biosynthesis pathways, mRNA expression related to aspartate aminotransferase (ASP5) and tyrosine aminotransferase (TAT), involved in the synthesis of phenylalanine and tyrosine, was significantly upregulated. Furthermore, DE-mRNAs in the scions of heterografted mao, resulting from RNA movement, also played an important role in these pathway changes. These mRNAs were associated with the upregulated expression of mRNAs encoding PRK, glyceraldehyde-3-phosphate dehydrogenase (GAPA), triosephosphate isomerase (TPI), shikimate dehydrogenase (SDH), ASP5, chalcone synthase (CHS), chalcone isomerase (CHI), naringenin 3-dioxygenase (F3H), and leucoanthocyanidin reductase (LAR) (Fig. 5B).

### Root application of kaempferol promotes the growth of *N. benthamiana*

**Figure 6:**
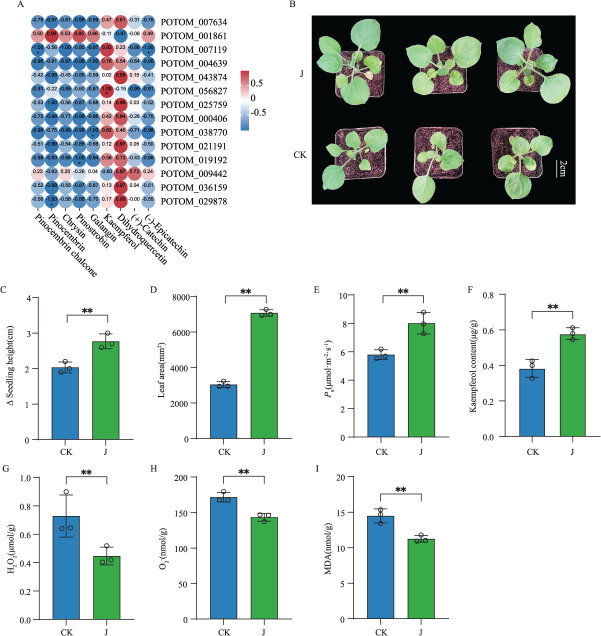
Analysis of key metabolites, and determination of phenotypic, growth and physiological indices in *N. benthamiana* under root application of kaempferol. (A) Correlation analysis between DE-mRNAs associated with mobile RNAs in the mechanistic diagram and differentially accumulated flavonoid metabolites; Darker circle colors indicate stronger correlations; red indicates positive correlations and blue indicates negative correlations; Pearson’s correlation coefficient was used for correlation analysis, and * indicates a significant correlation. (B) Phenotypes of *N. benthamiana* after root application of kaempferol; J and CK indicate the treatment and control groups, respectively. (C) Seedling height increment. (D) Leaf area. (E) Net photosynthetic rate. (F) Kaempferol content. (G) Hydrogen peroxide (H₂O₂) content. (H) Superoxide anion (O₂^−^) content. (I) Malondialdehyde (MDA) content.

Metabolites represent the ultimate functional products of gene expression and metabolic regulation. To validate potential key metabolites that may be influenced by mobile RNAs, correlation analyses were first performed between DE-mRNAs associated with mobile RNAs in the mechanistic diagram and DAMs in the flavonoid biosynthesis pathway. The results revealed a significant positive correlation between the expression level of *POTOM_056827* and kaempferol content (Fig. 6A). *POTOM_056827* was annotated as an aspartate aminotransferase, and its upregulation may promote the availability of phenylalanine, thereby indirectly enhancing phenylalanine-dependent flavonoid biosynthesis and ultimately facilitating kaempferol accumulation. Based on these findings, kaempferol was identified as a key metabolite potentially associated with mobile RNAs, and its regulatory role in plant growth was further examined by root application in *Nicotiana benthamiana*, a dicotyledonous plant. Subsequently, seedling height increment, leaf area, net photosynthetic rate, and the contents of kaempferol, H₂O₂, O₂^−^, and MDA were measured in kaempferol-treated plants. The results showed that kaempferol treatment significantly increased seedling height increment and leaf area (Fig. 6B–D), as well as net photosynthetic rate and leaf kaempferol content (Fig. 6E and F), while significantly reducing the contents of H₂O₂, O₂^−^, and MDA (Fig. 6G–I). These results indicate that root-applied kaempferol alleviates ROS-induced damage in *N. benthamiana*, thereby enhancing net photosynthetic capacity and promoting plant growth.

## Discussion

### Methods that rely less on single nucleotide polymorphisms (SNPs) are more suitable for the simultaneous identification of mobile mRNAs and lncRNAs

With the advancement of omics technologies, an increasing number of rootstock–scion mobile RNAs have been identified across various graft combinations. Currently, methods for the systematic identification of rootstock–scion mobile RNAs are primarily categorized into two types. The first type relies mainly on SNP markers. This method typically requires deep whole-genome resequencing of both rootstock and scion first to correct SNPs and insertions/deletions (INDELs) relative to the reference genome as much as possible. Subsequently, the identification of mobile RNAs can be performed using approaches similar to Li *et al.* [28]. This method has been successfully applied in studies by Liu *et al.* and Wang *et al.* [29, 30], and its success rates have been verified by RT-qPCR to reach 78.75% and 88.2%, respectively. While this method offers high accuracy, it involves large data volumes, complex operations, and relatively high costs. Moreover, its accuracy depends on SNP density; if the density is low, the loss of mobile RNAs may occur when using short-read transcriptome sequencing data for identification. The second type relies less on SNPs. This method, which is based on a high-quality reference genome, mainly uses software such as STAR or HISAT2 to achieve the identification of mobile RNAs. It avoids strong reliance on SNPs and allows a certain number of base mismatches. While effectively distinguishing reads derived from rootstock and scion, this method simplifies the identification workflow and reduces research costs. It has also been widely applied in the identification of rootstock–scion mobile RNAs. For instance, Li *et al.* [31] identified mobile sRNAs as well as mobile mRNAs in soybean and common bean. Wu *et al.* [32] simultaneously identified mobile lncRNAs and mRNAs between soybean and dodder. To date, few studies have systematically identified both mobile mRNAs and mobile lncRNAs between rootstocks and scions in woody plants. Considering the high heterozygosity, large genome size of woody plants, and low interspecific conservation of lncRNAs, the second method mentioned above was adopted for the identification of mobile RNAs. Validation of the mobile RNA identification results not only yielded full-length mobile mRNAs (both upwardly and downwardly mobile) but also full-length downwardly mobile lncRNAs. In particular, the 85% success rate verified by RT-qPCR further confirms the accuracy of this identification method. Studies have shown that some lncRNAs lack polyadenylation (polyA) tails [33]. Therefore, in this study, a strand-specific library construction method with rRNA depletion based on next-generation sequencing (NGS) was employed to comprehensively capture information on mobile lncRNAs. This method offers high throughput and low cost; however, its short read length leads to the loss of specific sequence information of mobile RNAs. In contrast, third-generation transcriptome sequencing can obtain full-length sequence information of the transcriptome, but it primarily relies on polyA-based library construction, which results in the loss of some polyA-lacking lncRNAs and is associated with higher costs. To obtain as comprehensive information of rootstock–scion mobile lncRNAs as possible in woody plants, this study employed next-generation sequencing. In future studies, we should integrate third-generation transcriptome sequencing to holistically evaluate the specific sequence information of mobile RNAs.

### lncRNAs exert extensive regulatory effects on mRNA expression

Studies have shown that lncRNAs are extensively involved in multiple biological processes in plants, including growth and development, maintenance of phosphorus homeostasis [15], regulation of flowering time [34], and responses to biotic stresses (e.g. pathogen infection) [35] and abiotic stresses (e.g. drought, high salinity, and extreme temperatures) [36–38]. This indicates that lncRNAs exert extensive regulatory effects on mRNA expression. Results of this study demonstrated that lncRNAs are associated with expression changes of mRNAs in almost all key pathways during rootstock–scion interaction, including pathways related to hormone synthesis, energy metabolism, and secondary metabolism. Furthermore, lncRNAs can move cross-tissue via the vascular system in grafted plants and may potentially regulate gene expression. These findings all reflect the extensive mRNA regulatory roles of lncRNAs, which are attributed to their diverse regulatory mechanisms: First, at the transcriptional level, lncRNAs can induce chromatin remodeling in the nucleus via histone methylation and acetylation modifications, thereby regulating the transcriptional activity of adjacent or distant mRNAs. They can also affect mRNA transcription by inducing promoter methylation of target mRNAs [39]. Second, at the post-transcriptional level, lncRNAs can bind to splicing factors to regulate alternative splicing of mRNAs, or interact with miRNAs to alleviate the inhibitory effect of miRNAs on their target mRNAs [13, 36]. In addition, lncRNAs can bind to proteins to regulate their subcellular localization, thereby participating in more extensive regulation of mRNA expression [40]. These regulatory mechanisms may also explain why the number of cis-target mRNAs was lower than that of trans-target mRNAs in this study. Although a large number of target mRNAs were predicted via lncRNAs, only a small subset showed significant changes in expression levels. Excluding factors such as differential mRNA screening criteria and false positives, this phenomenon may be associated with the high tissue specificity of lncRNA functions. The samples used for transcriptome sequencing in this study were a mixture of leaf and phloem tissues, which may have diluted the expression levels of some target mRNAs. Moreover, some lncRNAs may exert weak regulatory effects, while the protein levels encoded by their target genes have already changed. This also suggests that we should integrate proteomics to further clarify the regulatory mechanisms of lncRNAs. In this study, only one lncRNA with a full-length transport sequence was successfully validated in woody plants. Whether other mobile lncRNAs are mobile in full length, and whether fragmentarily mobile lncRNAs possess regulatory functions, require focused investigation in future work.

### Active transport mechanisms exist for mobile RNAs between rootstock and scion

The transport mechanism of mobile RNAs between rootstock and scion has long been a focus of research, as it can provide a solid theoretical basis for precision molecular breeding. In this study, we found that the abundance of downwardly mobile RNAs (both mRNAs and lncRNAs) was positively correlated with their mobility, whereas upwardly mobile RNAs showed no such correlation. This finding is both consistent with and contradictory to the study by Calderwood *et al.* [41], who demonstrated that in *Arabidopsis* grafting experiments, mRNAs with higher abundance in the donor were more likely to be mobile. It is also both consistent with and contradictory to the studies by Wang *et al.* and Xia *et al.* [30, 42], who both showed that the abundance of mobile mRNAs in the donor was not associated with their mobility. This discrepancy may be related to the distance of RNA movement. Since different mobile RNAs function in different tissues and upward movement involves a longer distance, the probability of degradation during movement increases, thereby affecting the correlation. The study by Yang *et al.* [43] also showed that the number of mobile mRNAs identified in micrografted grapevines was greater than that in field-grafted grapevines, possibly because field-grown grapevines are taller, and mobile mRNAs are more likely to be degraded during long-distance transport. In future studies, mobile RNAs should be identified in specific tissues to clarify the relationship between mobile RNAs and their abundance. The general consensus from current research is that RNA-degrading enzymes are absent in the phloem [44]. Thus, how these mobile RNAs are degraded remains an open question that requires further investigation. Whether the correlation between downwardly mobile RNAs and abundance is determined by intrinsic properties of the RNAs themselves or by the specific grafting position needs to be verified in future experiments using 107 as scion and mao as rootstock. In this study, differential mRNAs associated with upwardly mobile mRNAs were mainly enriched in amino acid metabolism-related pathways, whereas target mRNAs of upwardly mobile lncRNAs were primarily enriched in energy metabolism pathways, suggesting that the movement of mobile RNAs between rootstock and scion is subject to a strict, actively regulated mechanism. First, the migration of mobile RNAs requires passage through plasmodesmata in the symplast to enter sieve tubes. The pore size of plasmodesmata may be a limiting factor, and this size is influenced by both plant developmental stage and growth environment, enabling plants to selectively transport mobile RNAs as needed [45, 46]. It has been suggested that specific motifs present in mobile RNAs can bind to certain proteins, which can both increase the pore size of plasmodesmata and guide the RNAs to their target tissues to exert functions [47, 48]. Methylation modifications on these motifs can also promote the migration of mobile RNAs [49, 50]. Given that both mobile mRNAs and mobile lncRNAs have relatively long sequence lengths and show the same relationship with abundance during upward and downward transport, we speculate that their transport mechanisms may be similar, although this requires further research to confirm.

### Flavonoids may play an important role in coordinating rootstock–scion growth

Flavonoids play roles in signal transduction, ROS scavenging, and photoprotection in plants [51, 52]. The results of this study indicate that flavonoids play an important role in promoting the growth of the mao scions in heterografted combinations. First, flavonoids exhibit antioxidant activity, enabling them to scavenge ROS in plant cells and reduce the production of ROS signals. ROS signaling plays a key role in the ABA response; its reduction can increase stomatal aperture, thereby promoting CO₂ uptake by plants [53, 54]. In *N. benthamiana* treated with root-applied kaempferol, the leaf kaempferol content increased, ROS levels decreased, and net photosynthetic rate improved, reflecting this key function of flavonoids. Second, flavonoids can reduce UV-induced damage to thylakoids [55, 56], which can provide sufficient ATP and NADPH for the dark reactions of photosynthesis. Coupled with the upregulation of mRNAs related to CO₂ fixation in the Calvin cycle pathway in heterografted mao scions, these effects collectively enhance plant photosynthesis. This not only provides more energy to promote plant growth but also supplies more precursors for flavonoid biosynthesis, thereby forming a positive feedback loop. In this loop, the downregulation of mRNAs related to tryptophan synthesis may be one of the reasons for the reduction in leaf auxin levels. The significance of reduced leaf auxin content may be to slow scion development and promote the better allocation of energy produced by the scion to the rootstock, allowing the rootstock to adapt to scion development. Changes in the expression of mRNAs related to energy metabolism pathways in the rootstock, influenced by mobile RNAs, also represent an adaptation to scion growth. The study by Song *et al.* [57] showed that cucumber scions can affect auxin levels in pumpkin rootstocks, thereby influencing adventitious root growth. Harrison *et al.* [58] also found that different scions varieties had varying effects on the root system architecture of the rootstock in different apple grafting combinations. Both findings reflect the coordinated growth of rootstock and scion. Studies have shown that flavonoids can affect the activity of proteins related to auxin polar transport, thereby regulating plant growth [59], which further underscores the important role of flavonoids in coordinating rootstock–scion growth. In addition, the expression of some mRNAs involved in the flavonoid biosynthesis pathway is affected by mobile RNAs between rootstock and scion. However, the role of mobile sRNAs in coordinating rootstock–scion growth, whether mobile metabolites are directly involved, and the mechanism of energy allocation between rootstock and scion, require focused attention in future studies. In this study, the functional validation of flavonoids was performed using herbaceous plants. Since the vascular system differs from that of woody plants, future studies are needed to apply kaempferol to woody plants and conduct long-term observations to more clearly elucidate its role in plant growth.

## Materials and methods

### Plant materials and experimental design

In March 2021, 1-year-old cuttings of poplar 107 (107) and *P. tomentosa* (mao) were planted in the nursery of Hebei Agricultural University. The cuttings were ~1 cm in diameter and 12 cm in length. In 2022, seedlings with uniform growth were selected and coppiced at ~20 cm above ground level. The coppiced basal parts, together with its root system, was then transplanted to the Mancheng District Forest Farm in Baoding for grafting. Four graft combinations were established using the bud grafting method: 107/107, mao/107, mao/mao, and 107/mao (scion/rootstock). In March 2023, the scions of all grafted seedlings were subjected to coppice treatment, and in July of the same year, tissue sample collection and the determination of various indicators were conducted. All seedlings were managed under normal irrigation conditions throughout the experiment.

*Nicotiana benthamiana* seedlings were grown in pots with a volume of ~250 ml under greenhouse conditions (25 ± 2°C, 16 hours light/8 hours dark photoperiod, 60–70% relative humidity). The growth substrate consisted of a 1:1:1 (v/v/v) mixture of garden soil, nutrient soil, and vermiculite. After full expansion of the cotyledons, seedlings were treated with a 50-μM kaempferol solution applied to the roots every 7 days at a volume of 3 ml per plant, while control plants received an equal volume of water. Routine irrigation was performed every 3 days with 10 ml of water. After 2 weeks of growth, various physiological and growth-related parameters were measured.

All treatments consisted of 10 biological replicates and were arranged in a completely randomized block design.

### Determination of growth and physiological indicators

Taking the scions of mao as the research objects, their growth and physiological indicators were determined. The growth indicators included seedling height and basal diameter. The net photosynthetic rate was measured at 9:00 a.m. using a Li-6400 photosynthetic apparatus. The soluble sugar content was determined using the anthrone colorimetric method: with glucose as the standard, soluble sugars were extracted with absolute ethanol, then reacted with anthrone-sulfuric acid reagent in a boiling water bath for color development. After rapid cooling, the absorbance was measured at 620 nm [60]. The soluble protein content was determined using the Coomassie Brilliant Blue G-250 method: a standard curve was prepared with bovine serum albumin, and after the leaf homogenate reacted with the dye, the absorbance was measured at 595 nm [61]. Auxin (IAA), CTK, gibberellins (GA), and ABA were determined using corresponding kits purchased from Quanzhou Ruixin Biological Technology Co., Ltd, with the Cat. No.: RXJ1401005PL, RXJ1401590PL, RXJ1401581PL, and RXJ1401520PL, respectively. The specific determination method refers to the kit instruction manual. All the above indicators were measured with three biological replicates, and the tissue samples were collected from the third to fifth mature leaves of the scion, counted from top to bottom.

*Nicotiana benthamiana* was used as the experimental material for the determination of growth and physiological parameters. Growth parameters included seedling height increment and leaf area (calculated using Lamina software). The measurement of net photosynthetic rate was conducted as described above. Kaempferol content was quantified using high-performance liquid chromatography (HPLC). The contents of hydrogen peroxide (H₂O₂), superoxide anion (O₂^−^), and malondialdehyde (MDA) were determined using commercial assay kits purchased from Suzhou Grace Biotechnology Co., Ltd, with catalog numbers G0168W48, G0116W48, and G0109W4, respectively, following the manufacturer's instructions. All measurements were performed with three biological replicates. Tissue samples were collected from the two to five fully expanded leaves from the apex downward. The total leaf area of these leaves was used to represent the leaf area of each biological replicate.

### Determination and analysis of widely targeted metabolomics

Leaf and phloem tissues from the upper, middle, and lower parts of mao scions were collected, mixed uniformly (with three biological replicates), and then entrusted to MetWare Biotechnology Co., Ltd for widely targeted metabolomics determination. First, lyophilized samples were weighed and subjected to low-temperature extraction using 70% methanol–water. After centrifugation, the supernatant was filtered through a 0.22-μm filter membrane for subsequent ultra-HPLC–tandem mass spectrometry analysis [62]. Metabolite qualitative identification was based on the company's in-house database, while quantitative determination was performed using the multiple reaction monitoring mode with internal standard method for calibration. Meanwhile, quality control samples were used throughout the experiment to monitor system stability. The MultiQuant software was employed to calculate the mass spectrometry peak areas, which were used to represent the relative contents of the corresponding metabolites [63]. The specific experimental operations and detailed parameters of the relevant software were implemented in accordance with the company's standard protocols.

DAMs were screened based on the following criteria: variable importance in projection (VIP) > 1 and |log₂(fold change)| > 0.585. PCA was used to evaluate the differences among samples from different groups, and the KEGG database was employed for metabolite annotation.

### Transcriptome determination and identification of mobile RNAs

The same tissues used for metabolomics analysis, along with phloem tissues from 107 rootstocks (~10 cm below the graft union, three biological replicates), were entrusted to Tiangen Biotech Co., Ltd for transcriptome sequencing. First, total RNA that passed quality control was subjected to rRNA depletion using the TIANSeq rRNA Depletion Kit (Cat. No.: NR101). Then, mRNA and lncRNA libraries were constructed using the TIANSeq Stranded RNA-Seq Kit (Cat. No.: NR103). The constructed libraries were sequenced on the Illumina platform, ensuring that the sequencing data volume for each sample exceeded 14.5 Gb. Finally, the filtered clean reads were aligned to the corresponding reference genomes using HISAT2 software [64, 65]. The reference genome for mao is available at: https://ftp.ncbi.nlm.nih.gov/genomes/all/GCA/018/804/465/GCA_018804465.1_PTv2/GCA_018804465.1_PTv2_genomic.fna.gz. The reference genome for 107 is available at: https://ftp.ncbi.nlm.nih.gov/genomes/all/GCA/015/852/605/GCA_015852605.2_ASM1585260v2/GCA_015852605.2_ASM1585260v2_genomic.fna.gz. The lncRNAs identified by both Coding Potential Calculator 2 (CPC2) [66] and Coding-Non-Coding Index (CNCI) [67] were selected as the final set of recognized lncRNAs, with the following criteria: length > 200 bp and number of exons ≥2. The expression levels of mRNAs and lncRNAs were quantified using the FPKM (fragments per kilobase of transcript per million mapped reads) values. Cis-target mRNAs of an lncRNA were defined as mRNAs located within 100 kb upstream or downstream of the lncRNA. Trans-target mRNAs were identified as mRNAs that showed a significant correlation with the lncRNA, with a Pearson correlation coefficient |*r*| ≥ 0.7 and a *P*-value <0.05. DE-mRNAs and differentially expressed lncRNAs (DE-lncRNAs) were screened using the following criteria: |log₂(fold change)| ≥ 1 and *P* < 0.05. Functional annotation of mRNAs was performed using the KEGG database. The functional roles of lncRNAs were further predicted based on the annotation results of their target mRNAs.

The identification of scion–rootstock mobile RNAs was performed with modifications based on the method described by [29, 31]. Briefly, the same strategy was applied to identify mobile mRNAs and mobile lncRNAs. Taking the RNAs transported from mao/107 to mao/107 (the underlined part indicates the tissue to be described) as an example: (i) dual-genome alignment and candidate read filtering. The clean reads from mao/107 and 107/107 sequencing were first aligned to the 107 and mao reference genomes, respectively, using HISAT2. Next, the pysam module of Python was used to extract the list of read names that failed to align to the 107 genome but aligned to the mao genome. (ii) Extraction of alignment information and quantitative analysis. Using samtools and awk, the corresponding reads were then extracted from the BAM files generated by aligning mao/107 and 107/107 reads to the mao genome. HTSeq was employed to quantify their expression levels based on the mao genome. (iii) Identification of mobile RNAs. RNAs with an expression level > 0 in mao/107 but no expression in 107/107 were considered candidate mobile RNAs, to further reduce false positives, the candidate mobile RNAs in mao/107 that also showed expression (>0) in mao/mao were defined as the final set of mobile RNAs. The same procedure was applied to identify RNAs moving upward in mao/107. The specific parameters for each software were set according to the standard of Tiangen Biotech Co., Ltd.

### RT-PCR, Sanger sequencing, and RT-qPCR

Reverse transcription of the RNA used for transcriptome sequencing was performed using the LnRcute lncRNA cDNA First-Strand Synthesis Kit (Cat. No.: KR202-02) from Tiangen Biotech Co., Ltd. PCR amplification was carried out with the 2× M5 HiPer plus Taq HiFi PCR mix (Cat. No.: MF002-Plus-10) from Mei5 Biotechnology Co., Ltd, using cDNAs samples pooled from three biological replicates of the same treatment. The primers used were listed in Table S26. PCR products were purified using the FlaPure Gel Purification Kit (Cat. No.: GE706). Subsequently, the purified fragments were ligated into a T-vector, followed by transformation into *Escherichia coli* competent cells. Single colonies were picked and sent to Youkang Biotechnology Co., Ltd for Sanger sequencing. qPCR was performed using the LnRcute lncRNA Quantitative Detection Kit (Cat. No.: FP402-02) from Tiangen Biotech Co., Ltd, with three biological replicates. The primers were provided in Table S27. *Actin* was used as the reference gene, and the relative expression levels were calculated using the 2^−ΔΔCt^ method [68, 69].

### Integrated analysis of transcriptome and metabolome

DE-mRNAs and DAMs in the mao scion were simultaneously subjected to KEGG enrichment analysis. DE-mRNAs and DAMs enriched in the same pathway were considered to be functionally linked, and the results were ranked based on the significance of mRNA enrichment. Pathway mechanism map between DE-mRNAs and DAMS were generated using Adobe Illustrator.

### Statistical analysis of data

The best reciprocal orthologous mRNAs identification was performed using TBtools software. SPSS software was employed for statistical analysis of data significance and correlation analysis. GraphPad Prism 10.1.2 was used to generate bar charts, scatter plots, and box plots. Venn diagrams were constructed and KEGG enrichment analysis was carried out using R software. DNAMAN software was utilized to align the Sanger sequencing results. Adobe Illustrator was applied for the assembly and optimization of all figures.

## Supplementary Material

Web_Material_uhag086

## Data Availability

The raw RNA-seq data have been deposited in the NCBI under accession number PRJNA1337898. All other data are included in the article and its supplementary files.
