# Supplementary material for: Integrated multi-omics analysis reveals the molecular mechanism underlying poplar 107 rootstock–mediated regulation of Populus tomentosa scion growth
Source: Hortic Res. 2026 Mar 11;13(7):uhag086. doi: 10.1093/hr/uhag086 (PMC13291916; doi:10.1093/hr/uhag086)
Supplement: Web_Material_uhag086 [file Web_Material_uhag086.zip › Supplemental Figure.docx]

Supplementary Fig. S1. DE-RNAs and KEGG enrichment of heterografted 107 rootstocks. A: Plot of the number of upregulated and downregulated DE-mRNAs. B: Plot of the number of upregulated and downregulated DE-lncRNAs. C: Venn diagram of target mRNAs of DE-lncRNAs and DE-mRNAs. D: Venn diagram of DE-lncRNAs with cis-regulatory roles and those with trans-regulatory roles. E: KEGG enrichment bar plot of all upregulated mRNAs and all upregulated mRNAs associated with DE-lncRNAs. F: KEGG enrichment bar plot of all downregulated mRNAs and all downregulated mRNAs associated with DE-lncRNAs.

Supplementary Fig. S2. Validation of the accuracy of scion-rootstock mobile RNAs by Sanger sequencing. "107" and "mao" denote the rootstock and scion, respectively, in the heterograft.
