# Supplementary figures and images for: Integrated multi-omics analysis reveals the molecular mechanism underlying poplar 107 rootstock–mediated regulation of Populus tomentosa scion growth
Source: Hortic Res. 2026 Mar 11;13(7):uhag086. doi: 10.1093/hr/uhag086 (PMC13291916; doi:10.1093/hr/uhag086)

A

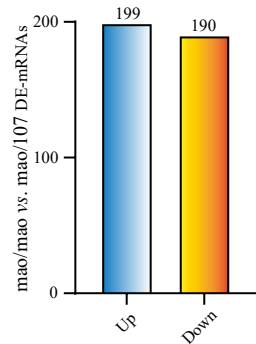

B

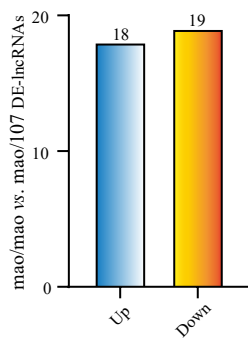

C

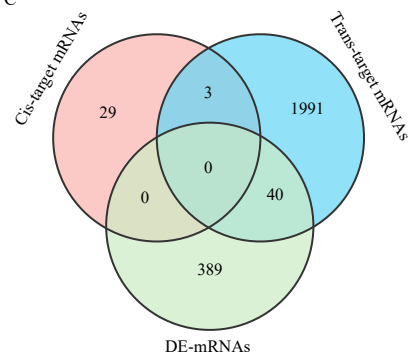

D

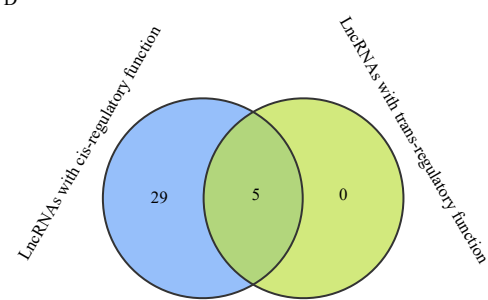

E

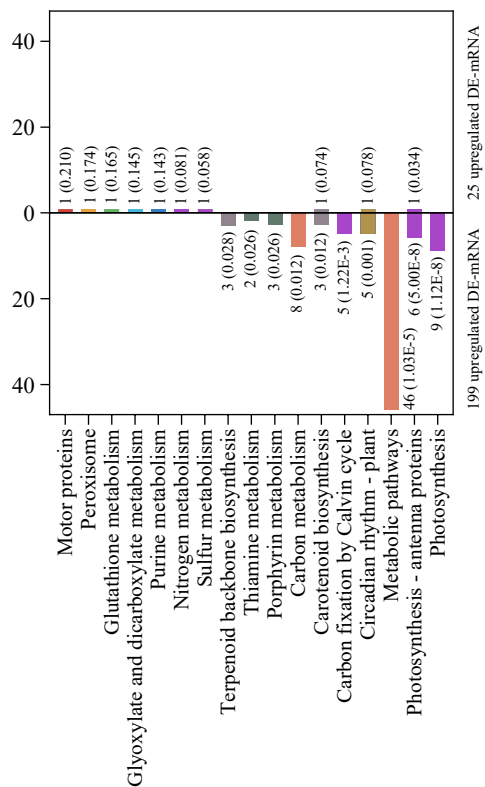

F

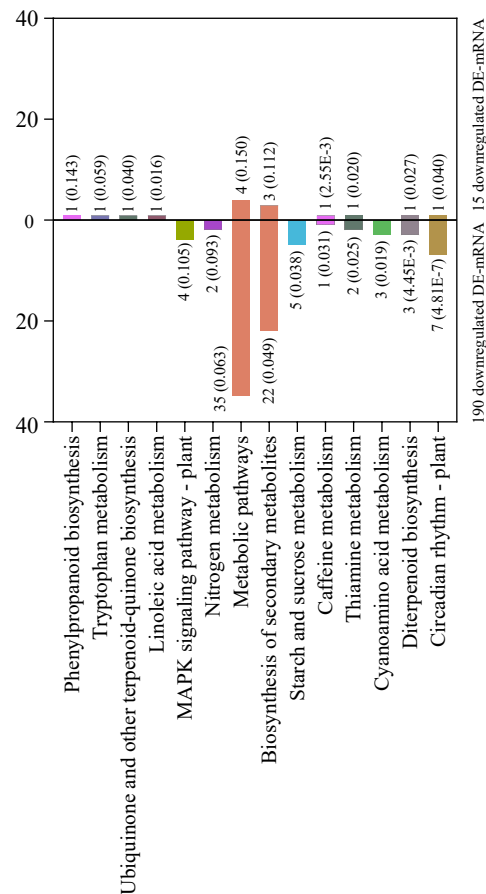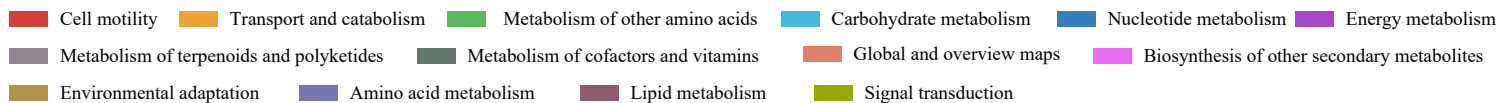

Supplement: Web_Material_uhag086 [file Web_Material_uhag086.zip › Supplementary Figure 1.pdf]
